# Skin and Syntax: Large Language Models in Dermatopathology

**DOI:** 10.3390/dermatopathology11010009

**Published:** 2024-02-14

**Authors:** Asghar Shah, Samer Wahood, Dorra Guermazi, Candice E. Brem, Elie Saliba

**Affiliations:** 1 Department of Dermatology, The Warren Alpert Medical School, Brown University, Providence, RI 02903, USA; asghar_shah@brown.edu (A.S.); samer_wahood@brown.edu (S.W.); dorra_guermazi@brown.edu (D.G.); 2 Section of Dermatopathology, Department of Dermatology, Chobanian and Avedisian School of Medicine, Boston University, Boston, MA 02118, USA; candice.brem@bmc.org; 3 Department of Dermatology, Gilbert and Rose-Marie Chagoury School of Medicine, Lebanese American University, Beirut 13-5053, Lebanon

**Keywords:** artificial intelligence, AI, LLM, large language models, dermatopathology

## Abstract

This literature review introduces the integration of Large Language Models (LLMs) in the field of dermatopathology, outlining their potential benefits, challenges, and prospects. It discusses the changing landscape of dermatopathology with the emergence of LLMs. The potential advantages of LLMs include a streamlined generation of pathology reports, the ability to learn and provide up-to-date information, and simplified patient education. Existing instances of LLMs encompass diagnostic support, research acceleration, and trainee education. Challenges involve biases, data privacy and quality, and establishing a balance between AI and dermatopathological expertise. Prospects include the integration of LLMs with other AI technologies to improve diagnostics and the improvement of multimodal LLMs that can handle both text and image input. Our implementation guidelines highlight the importance of model transparency and interpretability, data quality, and continuous oversight. The transformative potential of LLMs in dermatopathology is underscored, with an emphasis on a dynamic collaboration between artificial intelligence (AI) experts (technical specialists) and dermatopathologists (clinicians) for improved patient outcomes.

## 1. Introduction

The field of dermatopathology has witnessed a remarkable evolution in its diagnostic tools and techniques, reflecting the continued pursuit of precision in understanding and treating skin disorders [1]. From the early days of visual examination to the utilization of advanced microscopy and molecular analyses, dermatologists and dermatopathologists have continually strived to enhance their diagnostic capabilities. In this era of rapid technological advancement [1], Artificial Intelligence (AI) and Large Language Models (LLMs)—a form of AI that is often used to recognize and generate text—are emerging as potent tools for improving diagnostic sensitivity and accuracy. These intelligent systems, trained on data to understand and generate human-like text, are poised to revolutionize dermatopathology by offering insights into the complexities of skin-related conditions [2]. This literature review explores the transformative potential of AI-driven LLMs in dermatopathology by tracing the historical evolution of diagnostic tools in this specialized field, highlighting advantages associated with the use of LLMs in dermatopathology, and describing real-world applications of this emerging technology. Moreover, implementation challenges are described to contextualize the applicability of the prospects of this technology. Through this investigation of the literature, we aim to unveil a promising future where human expertise collaborates harmoniously with AI to advance the diagnosis and treatment of dermatological conditions.

## 2. Background

The history and development of AI in medicine has traced a long journey, shaping healthcare in profound ways throughout the years. Beginning with the early stages of simulating human decision-making in the 1950s, AI’s evolution has marked significant epochs, from expert systems in the 1980s to the data-driven era of machine learning and deep learning in the 2010s [3]. In this landscape, Natural Language Processing (NLP) has emerged as a pivotal force, aiming to bridge the gap between medical text data and actionable insights [4]. NLP works by utilizing complex algorithms and linguistic models to decipher the intricacies of human language. It enables computers to not only recognize words but also comprehend context, syntax, semantics, and subtle nuances, which may not be picked up by human observers, that are inherent in communication [5]. This technology has revolutionized medical practices by enabling the extraction of vital information from unstructured texts, such as clinical notes and research papers. This information, in turn, is then more readily accessible for analysis. Through NLP, the previously untapped wealth of information contained within these texts becomes a valuable resource for healthcare professionals and stakeholders [6]. 

In more recent times, the emergence of LLMs, a form of advanced AI systems capable of processing and generating human-like language based on extensive training on datasets, has propelled NLP to unprecedented heights. In general, LLMs are founded on a neural network architecture that can understand and produce human-like text. These models analyze and generate language, enabling them to perform various language-related tasks such as text-generation, summary of inputted text, and answering of questions.

These LLMs, exemplified by commercially developed models built on big data, like GPT-4 and Bard, possess the remarkable ability to not only “understand” but also generate coherent and contextually appropriate narratives. Their potential applicability to specialized medical fields like dermatopathology is profoundly promising [2,7]. 

Dermatopathology, a subspecialty of dermatology and pathology, focuses on diagnosing skin conditions using the histopathologic assessment of skin tissue samples in concert with a holistic appraisal of clinical examination and history. It often involves deciphering pathologic features and patterns within the collected samples [8]. The application of LLMs in dermatopathology may revolutionize the way in which dermatopathologists analyze complex dermatological texts. These language models, if trained and validated on relevant materials, may assist experts, through analyzing the content of textual inputs, in comprehending the nuances of clinical notes, research papers, and pathology reports, aiding in more accurate diagnoses and informed treatment planning [9]. The complexity of dermatopathology cases has been increasing [10], and among 60 surveyed consultants in the UK, almost one-quarter reported complaints or serious incidents related to delayed dermatopathology reporting [11]. LLMs may be able to help improve dermatopathologists’ productivity and reduce reporting times. 

The dynamic interplay between AI, NLP, and specialized applications like dermatopathology underscores the transformative nature and potential of AI technology in healthcare [12]. It foreshadows an era of data-driven precision and enhanced medical insights. By harnessing the power of NLP and LLMs, dermatopathologists can extract crucial information from pathology reports and other clinical documentation and translate it into actionable knowledge, ultimately leading to improved patient care and outcomes.

## 3. Advantages of LLMs in Dermatopathology

### 3.1. Automated Reporting: Revolutionizing the Generation of Pathology Reports

One of the remarkable advancements that the integration of LLMs brings to the field of dermatopathology is the potential for automating the process of generating pathology reports. While we acknowledge the need for further development and testing to reach this endpoint of automated reporting, it is important to note that this generation, similar to most other AI technologies, would best serve patients if developed, supervised, and performed in conjunction with practitioners. 

Traditionally, dermatopathologists spend a significant amount of time meticulously examining skin tissue samples as slides under a microscope and crafting detailed reports that capture their impressions and findings [13]. This process is not only time-consuming but also demands a high level of specialized expertise.

With the advent of LLMs, the landscape is changing. These language models possess an unparalleled capacity to understand medical jargon, contextual nuances, and technical language. By feeding these models with relevant data and observations from dermatopathological studies, it becomes possible to develop a system that can automatically generate comprehensive and accurate pathology reports. Clinical data are often in unstructured free-text formats; NLP technologies allow for the extraction of key information, linking back to information from histopathological slides [14]. For example, one study demonstrated the potential of using LLMs for diagnosing breast cancer patients, and it underscores the adaptability of these models for extracting clinical information in varied research contexts [15].

This automation could significantly reduce the time and effort expended by dermatopathologists on report creation, allowing them to focus more on the critical aspects of analysis and diagnosis [6]. Another potential benefit of AI driven pathology reporting, akin to other benefits of AI in healthcare, includes improving the efficiency of the workforce [16]. Given the nationwide shortage of dermatologists in the US, this technology may alleviate some of this burden through reducing inefficiency and assisting dermatopathologists with writing.

### 3.2. Continual Learning: Empowering LLMs to Assimilate and Provide Updated Medical Knowledge

The dynamic nature of medical knowledge necessitates that healthcare professionals stay abreast of the latest research, discoveries, and diagnostic methods. In dermatopathology, this requirement is particularly relevant due to the ever-evolving understanding of skin disorders. LLMs, with their capacity for continual learning, offer a transformative solution to this challenge.

LLMs can be designed to continuously assimilate new medical data, research papers, and clinical studies related to dermatopathology [17]. This enables them to stay up-to-date with the latest advancements and refine their understanding of the field over time. Consequently, when assisting dermatopathologists in analysis or generating reports, LLMs can provide insights that are not only informed by historical data but also enriched by the most recent medical knowledge. This integration of updated information into their analyses could substantially enhance the accuracy of diagnoses and treatment recommendations.

Additionally, users of LLMs like ChatGPT can also create and share custom GPTs to handle specific tasks [18]. If a dermatopathologist desires a GPT that can speak like a dermatopathologist and write notes like a dermatopathologist, it can be realized through instructions and by feeding the LLM with example notes in a desired formatting. The response can include a note template for a particular diagnosis. This custom dermatopathologist GPT can then be shared with all users in the GPT Store. Custom GPTs can also have their source knowledge fine-tuned based on particular relied-upon textbooks in dermatopathology, generating responses that are not clouded by other source material on the internet.

### 3.3. Patient Education: Bridging the Gap by Simplifying Complex Dermatopathological Concepts

Effective communication between dermatopathologists and patients is pivotal for successful treatment outcomes [19]. However, conveying intricate dermatopathological concepts to patients who lack medical backgrounds can be challenging. LLMs possess a unique potential to bridge this gap by simplifying complex medical terminology into understandable language [20].

These language models can be leveraged to translate the technical aspects of pathology reports into patient-friendly explanations. A recent cross-sectional study evaluated ChatGPT’s ability to reword dermatopathology reports into patient-friendly language. Most of the study subjects—including dermatology residents, dermatology fellows, board-certified dermatologists, and board-certified dermatopathologists—concluded that ChatGPT’s reworded reports were mostly complete, accurate, understandable, and unlikely to cause harm to patients [21]. Patient-friendly reports can supplement a physician-guided explanation to help patients comprehend their diagnoses, treatment options, and prognoses more comprehensively [22]. 

Moreover, LLMs can generate educational materials, such as brochures or online resources, that provide visually engaging and simplified explanations of various skin conditions [23]. By enhancing patient education and understanding, LLMs contribute to informed decision-making and facilitate a more collaborative approach to healthcare.

In conclusion, the incorporation of LLMs into dermatopathology holds transformative potential across various dimensions. From automating report generation to facilitating continuous learning and improving patient education, these language models are poised to reshape how dermatopathology is practiced. As LLMs become more integrated into healthcare workflows, they have the capacity to streamline processes, enhance diagnostic accuracy, and ultimately elevate the standard of care in dermatopathology.

## 4. Case Studies and Real-World Applications

This section delves into the practical applications of LLMs in the field of dermatopathology, showcasing their potential in various real-world scenarios (Table 1).

### 4.1. Diagnostic Support: How LLMs Can Assist in Identifying Rare or Atypical Presentations

One of the significant challenges in dermatopathology is diagnosing rare or atypical cases. LLMs, with their vast capacity to process and understand medical literature, have emerged as valuable tools in this context. These models can efficiently analyze a wide range of medical texts, providing insights that aid dermatopathologists in identifying and understanding uncommon skin conditions [24]. For instance, LLMs can swiftly review relevant case studies, research papers, and clinical notes to offer comprehensive information, enabling medical professionals to make more accurate diagnoses. Nonetheless, certain rare diseases within the domain of dermatology exhibit an exceedingly low case count, accompanied by an insufficient availability of specimens. This scarcity of data poses a substantial hurdle in furnishing machine learning algorithms with the requisite training, thereby constituting a significant obstacle for the advancement of AI in the field of dermatology [25]. While current LLMs can attempt to provide a report based on histopathologic imaging, they are limited with respect to accuracy [26]. There is a need for further validation on larger and more diverse datasets.

Multimodal LLMs can improve diagnostic support by drawing multiple sources of data input including clinical images, dermoscopic images, histopathological images, and text. The term multimodal refers to having the ability to engage with multiple modes of input rather than being unimodal, or one mode. One dedicated dermatological, multimodal LLM, SkinGPT-4, can diagnose common skin lesions based on clinical images and text descriptions provided by patients. The authors of the study indicated that several limitations exist, including a potential lack of trust from patients, which may result in an incomplete description provided to the LLM. Physicians can leverage their human connection with patients to facilitate adequate history-taking [27]. Additionally, the LLM does not currently support dermoscopic and histopathological images—meaning diagnoses would not be based on congruence between clinical examination, dermoscopy, and histopathological findings. 

### 4.2. Research: Accelerating Literature Reviews and Hypotheses Generation Using LLMs

The conventional approach to literature reviews for research purposes can be time-consuming and labor-intensive. LLMs offer an innovative solution by quickly comprehending and summarizing extensive medical texts, thus significantly accelerating the initial stages of research [24]. These models can assist researchers in generating hypotheses by analyzing existing data and proposing potential research directions. By leveraging LLMs in this manner, researchers can streamline the initial stages of their projects and focus more on in-depth analysis and experimentation [28].

### 4.3. Teaching and Training: LLMs as a Tool for Educating Novice Dermatopathologists

Training aspiring dermatopathologists presents a multifaceted challenge, demanding exposure to a diverse array of cases for the cultivation of diagnostic expertise. In this regard, LLMs from the Generative Pre-trained Transformers (GPT) series emerge as invaluable assets within the realm of medical education. These models assume a pivotal role by functioning as interactive educational tools [29,30]. By furnishing contextual information, elucidations, and simulated patient case studies, LLMs enrich the learning journey of budding dermatopathologists, adeptly bridging the gap between theoretical understanding and practical application. For instance, the integration of LLMs can profoundly reshape medical curriculum design, teaching methodologies, tailored study plans, learning resources, and student evaluations, ultimately elevating students’ proficiency and knowledge [31]. These models offer innovative resources for students, including interactive learning materials and simulated patient case studies. Additionally, LLMs can contribute to the evolution of assessment methods by providing insights into more effective student evaluations. By reshaping these fundamental aspects of medical education, LLMs play a pivotal role in enhancing students’ proficiency and knowledge, fostering a dynamic and comprehensive learning experience [31].

Yet, this transformative potential also prompts a critical examination of the complexities associated with such integration. Addressing concerns like algorithmic biases, undue reliance, plagiarism, dissemination of misinformation, disparities, privacy infringements, and copyright issues in the context of medical education is imperative. As we navigate the shift from an information-centric educational paradigm to one underscored by AI, a comprehensive understanding of both the prospects and challenges posed by LLMs in medical education becomes paramount. In this light, this paper not only offers insights into the potential of LLMs but also delves into the potential pitfalls. Our analysis serves as a foundational resource, informing forthcoming recommendations and best practices within the domain, thereby fostering the responsible and efficacious incorporation of AI technologies in medical education [31].

It is important to note that LLMs also suffer from producing some responses that support race-based medicine, which can confound results. One study showed that LLMs such as ChatGPT and Bard indicated that a Black skin is thicker than a White skin and that Black patients may have a higher pain tolerance than white patients [32]. Results such as these may find a way to perpetuate bias in decision-making.

In conclusion, this section sheds light on the diverse and valuable applications of LLMs in the realm of dermatopathology. By assisting in diagnosis, accelerating research, and enhancing education, LLMs have the potential to reshape the way professionals approach challenges in this field. However, it is essential to consider the ethical implications and continue refining the integration of LLMs to ensure their responsible and effective utilization in healthcare and education.

## 5. Discussion

The integration of LLMs into medical education, while promising, presents a set of intricate challenges and limitations that warrant a careful consideration.

### 5.1. Bias and Ethics

A central challenge surrounding the development of AI technologies revolves around the potential biases ingrained within the training data. Ensuring fairness and equity necessitates that training sets are sourced from a wide spectrum of patient populations, encompassing diverse skin tones and ethnic backgrounds [31]. Furthermore, the manner in which data are annotated demands attention; distinguishing between biopsy-proven cases and those labeled based solely on clinical examination or images is essential. Ethical considerations around the sourcing of training sets should also be attended to. Addressing these issues is imperative to avoid perpetuating disparities and to uphold the ethical foundation of medical education.

### 5.2. Data Privacy

The incorporation of LLMs raises valid concerns regarding the security and confidentiality of patient data [27]. Safeguarding sensitive information is paramount, necessitating robust data protection measures. Striking a balance between educational enhancement and patient privacy is pivotal, necessitating a comprehensive approach that adheres to regulatory guidelines and accepted ethical standards. Potential strategies to mitigate privacy concerns include the following: data sanitization, restricting user prompt lengths, and knowledge unlearning. These concerns should be addressed, and they constitute a barrier to the implementation of LLMs in dermatopathology, and health care more broadly [33].

### 5.3. Dependence vs. Assistance

Achieving an optimal equilibrium between leveraging AI support and upholding the significance of manual expertise represents a delicate endeavor. Positioning LLMs as collaborative tools that work alongside dermatopathologists, rather than as standalone entities, requires careful oversight. Designing systems that facilitate expert input and human decision-making is crucial for nurturing the growth of both learners and the technology itself.

### 5.4. Technical Limitations

While LLMs hold transformative potential, it is essential to acknowledge their current scope and limitations in the realm of medical education. The existing body of studies and evidence predominantly resides in the early stages, necessitating ongoing research and validation. Understanding the capabilities and boundaries of LLMs will foster informed decision-making and facilitate the gradual integration of these technologies into medical educational practices [28]. While LLMs like GPT and Bard are multimodal, they are not yet adequately developed for analyzing images like histopathological slides and scans. 

In navigating these challenges and limitations, a holistic and multidisciplinary approach is fundamental. By actively addressing biases, embracing ethical frameworks, safeguarding patient data, fostering collaboration, and comprehending the technical parameters, the responsible utilization of LLMs in medical education can be effectively realized. This proactive stance positions educators, professionals, and stakeholders to harness the transformative potential of LLMs while ensuring that their integration remains ethical, equitable, and aligned with the core tenets of medical education.

## 6. Future Directions

### 6.1. Interdisciplinary Collaboration

The integration of LLMs with other AI tools, particularly image recognition, presents a promising avenue for improving diagnostic accuracy in various medical domains, including dermatopathology. By combining the linguistic capabilities of LLMs with the visual analysis prowess of image recognition systems, a comprehensive understanding of both textual and visual medical data can be achieved. This synergistic approach holds the potential to revolutionize diagnostic processes by providing clinicians with a holistic view of patient cases, aiding in the accurate interpretation of medical images alongside clinical narratives [31].

Harnessing large datasets of pathology slides for training and validation will further strengthen this collaboration. LLMs can assist in extracting meaningful insights from vast amounts of textual data present in medical reports and the academic literature, while image recognition tools can leverage extensive image datasets to enhance pattern recognition and the identification of nuanced visual markers associated with various conditions. The collaboration between LLMs and image recognition, thus, augments the precision and efficiency of medical diagnoses, fostering a new era of data-driven interdisciplinary collaboration in healthcare.

### 6.2. Personalized Medicine 

LLMs offer the potential to revolutionize personalized medicine by providing clinicians with comprehensive patient-specific insights [28]. Through analyzing patient medical records including genetic data and treatment histories, LLMs can generate tailored recommendations, treatment plans, and prognoses. This personalized approach takes into account an individual’s unique medical history, genetic makeup, lifestyle factors, and even their responses to previous treatments. By processing and synthesizing vast amounts of patient data, LLMs enable clinicians to make more informed decisions, offering precision in diagnosis and treatment strategies that were previously unattainable. Furthermore, LLMs can facilitate patient education and engagement by generating easily comprehensible summaries of complex medical information. Patients can access detailed explanations of their conditions, potential treatment options, and associated risks, empowering them to actively participate in their healthcare decisions. The integration of LLMs into personalized medicine not only enhances patient outcomes but also streamlines clinical workflows by providing clinicians with timely and accurate insights tailored to each patient’s unique circumstances.

### 6.3. Expansion to Other Pathology Sub-Disciplines 

While the primary focus of this paper is on dermatopathology, the potential applications of LLMs extend far beyond this single sub-discipline [34]. As LLMs continue to evolve, their adaptable nature allows for an integration into a wide range of pathology domains. By training LLMs on diverse datasets from various sub-disciplines, these models can be equipped to interpret and analyze a plethora of medical content [35]. From identifying cellular structures and patterns in histopathology slides to analyzing radiological images, and even genomics data, LLMs can offer valuable insights across the entire spectrum of medical disciplines.

This expansion and its potential utility for improving patient outcomes necessitates collaboration between medical experts, data scientists, and AI researchers to tailor LLMs to the unique demands of each pathology sub-discipline. The integration of LLMs in diverse areas of pathology not only enhances diagnostic accuracy but also accelerates research, aids in data interpretation, and contributes to the overall advancement of medical science. As LLMs continue to demonstrate their versatility and potential, their integration into multiple pathology sub-disciplines holds the promise of transforming the landscape of medical diagnosis and treatment on a global scale.

## 7. Guidelines for Implementation

**Figure 1:**
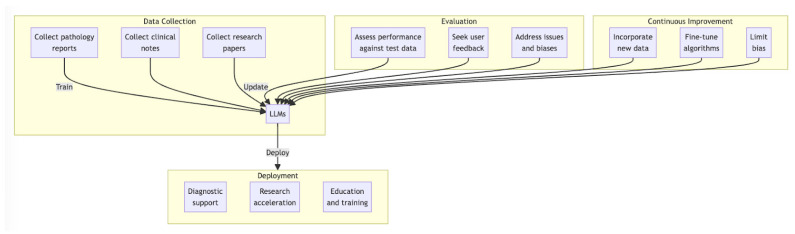
Proposed workflow for deployment of LLMs for dermatopathology.

Incorporating LLMs into the field of dermatopathology requires a structured approach to ensure their effective and trustworthy utilization. This section outlines essential guidelines for the successful implementation of LLMs in the medical community (Figure 1).

### 7.1. Training and Onboarding: Ensuring Understanding and Trust

The integration of LLMs into dermatopathology begins with a strong emphasis on training and onboarding. Medical professionals need to comprehend the capabilities and limitations of these models to trust their utility. Achieving this requires a commitment to transparency and education. It is imperative to avoid treating LLMs as full “black-box” models [36]. Instead, we should provide healthcare practitioners with insights into how LLMs function, their underlying algorithms, and the processes through which they generate responses. This transparency fosters trust and empowers healthcare providers to harness LLMs effectively in their clinical practice [37].

Moreover, data quality plays an instrumental role in the training and performance of LLMs. To ensure the reliability of these models, it is essential to curate high-quality, unbiased data for their training [37]. The datasets should be comprehensive, diverse, and representative of the dermatopathological cases encountered in real-world scenarios. Addressing biases in data collection and annotation is paramount to mitigating any inherent biases that may emerge in LLM outputs. Datasets used to train LLMs and custom GPTs should use clinical, dermoscopic, and histopathological images from patients of all skin types.

### 7.2. Ongoing Evaluation: Continuous Assessment of LLMs

The implementation of LLMs in dermatopathology does not conclude with their initial deployment. Continuous evaluation is imperative to maintain their accuracy and relevance [36]. Healthcare institutions should establish mechanisms for the periodic assessment of LLMs’ performance, scrutinizing their outputs against real-world cases and expert evaluations. Regularly updating the LLMs with new data and emerging medical insights ensures that they evolve alongside the field of dermatopathology. The implementation of LLMs should not be viewed as a one-time endeavor but rather an ongoing commitment to improvement. This necessitates a dynamic feedback loop involving healthcare practitioners, data scientists, and machine learning experts [38]. Regularly seeking input from dermatopathologists and other stakeholders ensures that LLMs remain aligned with the evolving needs of the medical community. Continuous improvement efforts should address issues, fine-tune algorithms, and incorporate user feedback to enhance the overall utility of LLMs in dermatopathology.

Ultimately, the successful implementation of LLMs in dermatopathology hinges on a foundation of transparency, high-quality data, continuous evaluation, and a commitment to ongoing quality improvement. By adhering to these guidelines, the medical community can harness the potential of LLMs while maintaining trust and accuracy in the diagnosis and treatment of dermatological conditions.

## 8. Conclusions

In summary, the integration of LLMs into the field of dermatopathology holds immense potential for transformative impact. This integration necessitates close collaboration between AI experts and dermatopathologists, emphasizing the importance of fruitful teamwork. The application of LLMs in dermatopathology promises not only increased diagnostic accuracy but also the potential for significantly improved patient outcomes and satisfaction. LLMs, with their ability to provide comprehensive and up-to-date information on dermatopathology findings, offer a powerful tool that can enhance healthcare delivery and elevate the quality of care provided to patients. Prior to the implementation of LLMs in dermatopathology, limitations including bias, accuracy, privacy concerns, and the inability to effectively analyze histopathologic images should be addressed. The future of LLMs includes interdisciplinary collaboration with image recognition to enhance diagnostic accuracy, the potential for personalized medicine by analyzing patient records and offering tailored recommendations, and the expansion of LLM applications beyond dermatopathology to various pathology sub-disciplines, promising to transform global medical diagnosis and treatment. Embracing these advancements responsibly and collaboratively can usher in a new era of dermatopathology that benefits both practitioners and those seeking medical guidance for skin-related conditions.

## Figures and Tables

**Table 1:** Potential applications of LLMs in dermatopathology.

Types	Examples
Diagnostic Support	Reviewing the literature for the histopathological findings in malignant proliferating trichilemmal tumors.
Research	Generating an outline based on ideas for a manuscript.
Teaching and Training	Generating questions based on a histopathological slide to check understanding.
Patient Education	Explaining a pathology report indicating nodular basal cell carcinoma to a patient.

## Data Availability

Not applicable.
